# Childhood suffering: hyper endemic echinococcosis in Qinghai-Tibetan primary school students, China

**DOI:** 10.1186/s40249-018-0455-y

**Published:** 2018-07-12

**Authors:** Xiu-Min Han, Qi-Gang Cai, Wei Wang, Hu Wang, Qiang Zhang, Yong-Shun Wang

**Affiliations:** 1 Hydatid Disease Clinical Research Institute, Qinghai Provincial People’s Hospital, Building No.1, Gonghe Road No.2, Chengdong district, Xining City, 810007 Qinghai Province China; 2 State Key Laboratory of Plateau Ecology and Agriculture, Qinghai Academy of Animal Science and Veterinary Medicine, Qinghai University, Xining, 810016 Qinghai Province China; 3 Qinghai Institute for Endemic Disease Prevention and Control, Xining, 811602 Qinghai Province China; 4 Public Health and Family Planning Commission, Xining, 810007 Qinghai Province China

**Keywords:** *Echinococcus*, Echinococcosis, High prevalence, Tibetan children, Qinghai, China

## Abstract

**Background:**

As part of an ongoing program that aims to use early detection and timely treatment to improve the control of echinococcosis, especially in younger age groups, we undertook a series of active surveys among Qinghai-Tibetan children in the Qinghai Province of Northwestern China in 2011 and 2012. The significant outcomes that resulted from this study emphasize the need to draw attention to echinococcosis, both alveolar echinococcosis (AE) and cystic echinococcosis (CE), so that policy development is promoted and suitable avenues for control are identified in the highly endemic areas on the Tibetan Plateau.

**Methods:**

A total of 19 629 primary school students, aged 6–18 years, with a dominant Tibetan background underwent abdominal ultrasound examination, and 86.4% of the compliant students donated 2–5 ml of venous blood for serological tests. All the abnormal ultrasound results were recorded. If identified as echinococcosis, the disease lesion was assessed according to the WHO-Informal Working Group on Echinococcosis (WHO-IWGE) classification for AE and CE. Among the surveyed students, the prevalence by school was compared among geo-locations, sex and age groups. The clinical image presentations were analyzed according to lesion number, size, the location in the liver and the classification stage. Statistical significance was set at *P*-value < 0.05 for comparisons among groups.

**Results:**

A total of 341 students (1.7%) were identified by ultrasound as having either CE (119, 0.6%) or AE (222, 1.1%). The highest prevalence rates of childhood AE cases occurred in the Tehetu (12.1%) and Moba (11.8%) townships in Dari County. There was a high seropositive rate (37.0%) and a heterogeneous distribution of cases, with a prevalence ranged from 0 to 12.1% for AE and 0–2.9% for CE. Moreover, the seropositive rate ranged from 0.7–45.1% across different schools.

**Conclusions:**

The high prevalence of echinococcosis in Qinghai-Tibetan primary school students reflects a lack of knowledge about *Echinococcus* spp. transmission. The combination of systematic education for children and regularly performed anthelmintic treatment for dogs could achieve the goal of sustainable hydatidosis control.

**Electronic supplementary material:**

The online version of this article (10.1186/s40249-018-0455-y) contains supplementary material, which is available to authorized users.

## Multilingual abstracts

Please see Additional file 1 for translations of the abstract into the six official working languages of the United Nations.

## Background

Cystic echinococcosis (CE) and alveolar echinococcosis (AE), caused by *Echinococcus granulosus* and *E. multilocularis*, respectively, are important human parasitic diseases worldwide. According to recent estimates, there are currently 60 million people at risk of infection and approximately 2–3 million cases of echinococcosis globally, with one-third of all cases occurring in children [1, 2]. Human CE poses an important global burden in terms of disability-adjusted life years (DALYs). Recent global figures also suggest that there are approximately 18 235 new cases of AE per annum, of which 91% (16 629) are diagnosed in China [3]. Based on the disability weights for hepatic carcinoma and the estimated age- and gender-specific incidence [3, 4], the median global cost of AE is 666 434 DALYs per annum [3]. However, these calculations are likely an underestimation of the actual costs due to the chronic status of AE that requires 30 years of daily treatment for most patients [5]. In the 1990s, the national investigation on the distribution of human parasites was implemented; analysis of hospital surgery records in China identified echinococcosis-endemic areas on the Tibetan Plateau (including parts of the Sichuan, Qinghai and Gansu provinces) and the Xinjiang and Ningxia autonomous regions. These surveys also showed that the prevalence of human AE was, in fact, higher than that of CE [2, 6], which was previously largely ignored because hospital records indicated that the number of AE cases was considerably less than that of CE cases [6]. These findings can be partially explained by the long asymptomatic period of AE, the lack of knowledge about the disease and the heavy economic burden that it presents for these communities. Furthermore, AE generally occurs in underdeveloped regions where the poor economic standards may limit the availability of diagnostic techniques and treatment. In 1994, after a national assessment of the major parasitic diseases in China, the Ministries of Health and Agriculture identified echinococcosis as a zoonotic disease with a major impact on the public health of rural populations in western China [7], particularly in the communities on the Tibetan Plateau [8]. To address these public health concerns, in 2006, the Chinese central government launched a program for echinococcosis control in 7 provinces/autonomous regions of western China (Qinghai, Xinjiang, Ningxia, Gansu, Inner Mongolia, Tibet and western Sichuan). This initiative aim is focused on the implementation of strategies to improve the prognosis of echinococcosis patients through early detection and timely treatment, especially in younger age groups. As part of this ongoing program, we describe the analysis of surveillance data collected from Qinghai-Tibetan children in the Qinghai Province, an area with a high prevalence of both AE and CE. Our aim is to draw attention to this highly endemic area for echinococcosis to promote policy development and to identify suitable avenues for control.

## Methods

### Study area

**Fig. 1:**
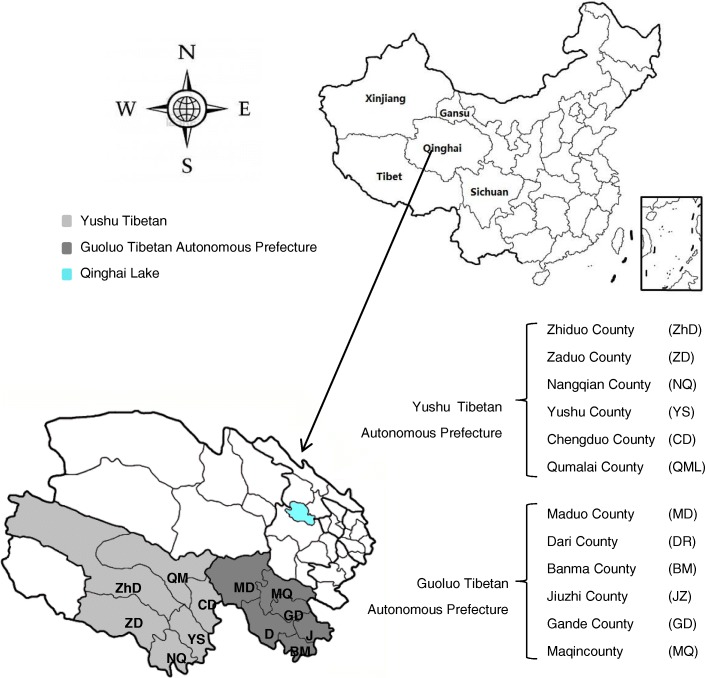
Investigated counties selected from the Yushu and Guoluo Tibetan autonomous prefectures

Six counties were selected from Yushu and Guoluo Tibetan Autonomous Prefectures (TAPs), which are situated in the south of the Qinghai Province, bordering the northwestern Sichuan Province (Fig. 1). The altitudes in this area range from 3600 to 4500 m above sea level. The total populations in Yushu and Guoluo are approximately 378 439 and 140 000 inhabitants, respectively. The area of both TAPs combined is 265106 km^2^ (Yushu: 188794 km^2^, Guoluo: 76312 km^2^). The inhabitants are mainly (96–98%) Chinese-Tibetan individuals. The religion (predominantly Buddhism), culture, socioeconomic structure, land-use pattern and population density in these counties are representative of typical rural Tibetan communities. The inhabitants are generally families living in poor conditions, and the main source of income is animal husbandry.

A survey was conducted among students in 65 schools from 58 townships in Yushu and Guoluo (18 schools and 47 schools, respectively, see Table 1). This survey represented 34% of the townships located in Yushu (schools from 15 out of 44 townships) and 95% of the townships in Guoluo (schools from 43 out of 45 townships). The surveyed schools were mainly boarding schools for primary, junior (years 1–3) and senior (years 4–6) education. While the survey also included some mixed-middle schools, it mainly involved primary school students. The primary schools in China are commonly composed of 6 grades (years 1–6).

**Table 1:** The distributions of morbidity/seropositive rates in school children, south Qinghai

Townships & counties	Demography				Ultrasound scan		Sero-test
	School code	# School type	Age (‾X ± SD)	size	CE/AE (prevalence, %)	Echinococcosis	Sero positive/children (prevalence, %)
Chengwen	1	CPS	11 ± 2.4	400	4/6(1.0/1.5)	10 (2.5)	14/394 (3.6)
Xiewu	2	MS	12 ± 2.2	277	0/1(0/0.4)	1(0.4)	2/276 (0.7)
Qingshuihe	3	BPS	13 ± 2.6	653	5/6 (0.8/0.9)	11 (1.7)	48/652 (7.4)
Zhaduo	4	BPS	12 ± 2.0	282	0/5 (0/1.8)	5 (1.8)	11/280 (3.9)
Chengduo County (CD)				1612	9/18 (0.6/1.1)	27 (1.7)	75/1602 (4.7)
Baizha	5	BPS	11 ± 2.6	123	0/0	0	3/123 (2.4)
Baizha	6	CPS	12 ± 2.6	180	1/0 (0.6/0)	1 (0.6)	3/180 (1.7)
Baizha	7	CPS	10 ± 1.9	839	1/0 (0.1/0)	1 (0.1)	16/839 (1.9)
Xiangda	8	BPS	11 ± 2.4	402	1/0 (0.2/0)	1 (0.2)	12/402 (3.0)
Nangqian County (NQ)				1544	3/0 (0.2/0)	3 (0.2)	34/1544 (2.2)
Yuegai	9	CPS	12 ± 2.5	833	1/8 (0.1/1.0)	9 (1.1)	60/833 (7.2)
Bagan	10	BPS	12 ± 2.5	395	3/3 (0.8/0.8)	6 (1.5)	22/395 (5.6)
Qumalai County (QML)				1228	4/11 (0.3/0.9)	15 (1.2)	82/1228 (6.7)
Jiegu	11	CPS	10 ± 2.2	1569	2/1 (0.1/0.06)	3 (0.2)	45/1545 (2.9)
Longbao	12	BPS	11 ± 2.2	409	1/0 (0.2/0)	1 (0.2)	31/409 (7.6)
Yushu County (YS)				1978	3/1 (0.15/0.05)	4 (0.2)	76/1954 (3.9)
Jieduo	13	BPS	10 ± 1.4	108	1/0 (0.9/0)	1 (0.9)	–
Sulu	14	BPS	11 ± 2.8	110	1/0 (0.9/0)	1 (0.9)	–
Zhaqing	15	BPS	11 ± 2.4	121	1/0 (0.8/0)	1 (0.8)	–
Zaduo County (ZD)				339	3/0 (0.9/0)	3 (0.9)	**–**
Duocai	16	BPS	11 ± 2.6	86	0/0	0	3/86 (3.5)
jiaji	17	BPS	12 ± 2.4	468	5/1 (1.1/0.2)	6 (1.3)	20/468 (4.3)
Jiaji	18	CPS	14 ± 1.3	199	0/0	0	12/199 (6.0)
Zhiduo County (ZhD)				753	5/1 (0.7/0.1)	6 (0.8)	35/753 (4.6)
Yushu TAP				7454	27/31 (0.4/0.4)	58 (0.8)	302/7081(4.3)
*P*-value					0.08/ 0.0003		
Zhiqin	19	BPS	11 ± 2.2	168	0/2 (0/1.2)	2 (1.2)	22/139 (15.8)
Sailaitang	20	MS	9 ± 1.6	224	0/2 (0.9)	2 (0.9)	15/196 (7.7)
Daka	21	BPS	10 ± 2.5	60	0/3 (0/5.0)	3 (5.0)	12/46 (26.1)
Dengta	22	BPS	10 ± 1.5	92	0/0	0	3/88 (3.4)
Duogongm	23	BPS	10 ± 1.7	187	2/4 (1.1/2.1)	6 (3.2)	36/187 (19.3)
Jika	24	BPS	11 ± 2.3	193	1/4 (0.5/2.1)	5 (2.6)	37/133 (27.8)
Jiangritang	25	BPS	10 ± 1.5	110	0/3 (0/2.7)	3 (2.7)	11/99 (11.1)
Makehe	26	BPS	10 ± 1.7	159	0/8 (0/5.0)	8 (5.0)	20/155 (12.9)
Yaertang	27	BPS	10 ± 1.8	77	0/0	0	6/64 (9.4)
Banqian	28	BPS	10 ± 1.5	81	0/0	0	5/78 (6.4)
Banma County (BM)				1351	3/26 (0.2/1.9)	29 (2.1)	167/1185(14.1)
Jimai	29	MS	10 ± 1.5	240	2/4(0.8/1.6)	6 (2.5)	31/226 (13.7)
Jimai	30	BPS	9 ± 1.3	443	1/17 (0.2/3.8)	18 (4.0)	106/430 (24.7)
Deang	31	BPS	11 ± 2.3	233	0/11^a^ (0/4.7)	11 (4.7)	36/156 (23.1)
Jianshe	32	BPS	10 ± 1.8	343	2/8 (0.6/2.3)	10 (2.9)	17/89 (19.1)
Manzhang	33	BPS	11 ± 2.4	267	5/6 (1.9/2.2)	11 (4.1)	45/195 (23.1)
Moba	34	BPS	10 ± 2.4	153	1/18 (0.6/11.8)	19(12.4)	22/106 (20.8)
Sangrima	35	BPS	10 ± 1.8	126	1/7 (0.8/5.6)	8 (6.3)	25/125 (20.0)
Shanhonke	36	BPS	10 ± 1.5	150	3/8 (2.0/5.3)	11(7.3)	39/145 (26.9)
Tehetu	37	BPS	11 ± 2.4	207	1/25 (0.5/12.1)	26(12.6)	57/154 (37.0)
Wosai	38	BPS	10 ± 2.2	194	1/1 (0.5/0.5)	2 (1.0)	27/139 (19.4)
Xiahongke	39	BPS	10 ± 1.4	105	3/2 (2.9/1.9)	5 (4.8)	24/99 (24.2)
Dari County^a^(DR)				2461	20/107(0.8/4.3)	127(5.2)	429/1864 (23.0)
Kequ	40	MS	10 ± 2.0	766	7/6 (0.9/0.8)	13 (1.7)	129/698 (18.5)
Ganglong	41	BPS	10 ± 1.5	248	2/1 (0.8/0.4)	3 (1.2)	41/245 (16.7)
Jiangqian	42	BPS	10 ± 1.6	185	2/1 (1.1/0.5)	3 (1.6)	34/183 (18.6)
Qingzhen	43	BPS	10 ± 1.7	478	8/5 (1.7/1.0)	13 (2.7)	109/466 (23.4)
Shangongm	44	BPS	9 ± 1.8	175	4/3 (2.3/1.7)	7 (4.0)	45/175 (25.7)
Xiazangke	45	BPS	10 ± 1.4	260	2/2 (0.8/0.8)	4 (1.5)	50/260 (19.2)
Xiagongma	46	BPS	11 ± 2.3	337	6/1 (1.8/0.3)	7 (2.1)	46/247 (18.6)
Gande County (GD)				2449	31/19 (1.3/0.8)	50 (2.0	454/2274 (20.0)
Baiyu	47	BPS	11 ± 2.3	527	11/6 (2.1/1.1)	17 (3.2)	52/187 (27.8)
Zhiqinsond	48	BPS	10 ± 1.8	319	0/0	0	8/107 (7.5)
Mentang	49	BPS	10 ± 1.6	216	1/0 (0.5/0)	1(0.5)	28/204 (13.7)
Suohurima	50	BPS	10 ± 1.6	341	2/1 (0.6/0.3)	3 (0.9)	70/330 (21.2)
Waeryi	51	BPS	10 ± 1.6	221	3/4 (1.4/1.8)	7 (3.2)	46/218 (21.1)
Wasai	52	BPS	11 ± 2.3	284	2/1 (0.7/0.4)	3 (1.1)	51/232 (22.0)
Jiuzhi County (JZ)				1908	19/12 (1.0/0.6)	31 (1.6)	255/1278 (20.0)
Dawu	53	MS	10 ± 1.9	955	7/7 (0.7/0.7)	14 (1.5	120/858 (14.0)
Dawu	54	BPS	11 ± 2.0	194	1/1 (0.5/0.5)	2 (1.0)	21/91 (23.1)
Dawu	55	CPS	10 ± 1.5	200	0/2 (0/1.0)	2 (1.0)	42/196 (21.4)
Dangluo	56	BPS	11 ± 2.1	248	1/6 (0.4/2.4)	7 (2.8)	34/192 (17.7)
Dongqingo	57	BPS	12 ± 2.5	114	0/0	0	18/74 (24.3)
Jiangqian	58	BPS	11 ± 1.9	77	0/1 (0/1.3)	1 (1.3)	15/63 (23.8)
Lajia	59	BPS	11 ± 2.4	803	0/0	0	106/557 (19.0)
Xiadawu	60	BPS	11 ± 2.3	191	1/0 (0.5/0)	1 (0.5)	60/133 (45.1)
Xueshan	61	BPS	11 ± 2.4	147	2/2 (1.4/1.4)	4 (2.7)	45/119 (37.8)
Youyun	62	BPS	12 ± 2.0	206	5/2 (2.4/1.0)	7 (3.4)	37/133 (27.8)
Maqin County (MQ)				3135	17/21 (0.5/0.7)	38 (1.2)	498/2416 (20.6)
Huashixia	63	BPS	12 ± 2.2	353	1/0 (0.3/0)	1 (0.3)	5/353 (1.4)
Huashixia	64	MS	12 ± 2.3	442	1/3 (0.2/0.7)	4 (0.9)	25/442 (5.7)
Machali	65	MS	10 ± 2.0	76	0/3 (0/3.9)	3 (3.9)	2/76 (2.6)
Maduo County (MD)				871	2/6 (0.2/0.7)	8 (0.9)	32/871 (3.7)
Guoluo TAP				12 175	92/191(0.8/1.6)	282 (2.3)	1835/9888(18.6)
*P*-value					0.0012/0.001		
Total				19 629	119/222(0.6/1.1)	341 (1.7)	2137/16969(12.6)

School code = all surveyed schools were labelled by numbers, of which the school name is presented as follow: 1, CD complete primary school (CPS); 2, The 2nd middle school (MS) of CD; 3, Qingshuihe centre boarding primary school (BPS); 4, ZD centre BPS; 5, Baizha centre BPS. 6, The 2nd CPS of NQ; 7, The 1st CPS of NQ; 8, Xiangda centre BPS. 9, The 2nd CPS of QML; 10, Bagan centre BPS; 11, The 3rd CPS of YS; 12, Longbao centre BPS; 13, Jieduo centre BPS; 14, Sulu centre BPS; 15, Zhaqing centre BPS; 16, Duocai centre BPS; 17, Jiga centre BPS; 18, ZhD CPS; 19, Zhiqin BPS; 20, BM MS for nationalities. 21, Daka Tibetan BPS; 22, Dengta BPS; 23, Duogongma BPS; 24, Jika BPS; 25, Jiangritang BPS; 26, Makehe BPS; 27, Yaertang BPS; 28, Banqian BPS; 29, DR MS for nationalities. 30, Jimai BPS; 31, Deang BPS; 32, Jianshe BPS; 33, Manzhang BPS; 34, Moba Tibetan BPS; 35, Sangrima BPS; 36, Shanghongke BPS; 37, Tehetu BPS; 38, Wosai BPS; 39, Xiahongke Tibetan BPS; 40, GD MS for nationalities. 41, Ganglong BPS; 42, Jiangqian BPS; 43, Qingzhen BPS; 44, Shanggongma BPS; 45, Xiazangke Tibetan BPS; 46, Xiagongma BPS; 47, Baiyu BPS; 48, Zhiqingsongduo BPS; 49, Mentang Daka Tibetan BPS; 50, Suohurima BPS; 51, Waeryi BPS; 52, Wasai BPS; 53, MQ MS for nationalities. 54, Dawu BPS; 55, The 1st CPS for Nationalities of MQ; 56, Dangluo BPS; 57, Dongqinggou BPS; 58, Jinagqian BPS; 59, Lajia BPS; 60, Xiadawu BPS; 61, Xueshan BPS; 62, Youyun BPS; 63, Huashixia BPS; 64, MD Tibetan MS; 65, MD MS for nationalities

*AE* Alveolar echinococcosis, *CE* Cystic echinococcosis, # CPS generally is located in county city; MS includes primary division, of which this survey only focused on. ^a^Here including one case with co-existence of CE/AE; TAP, Tibetan Autonomous Prefecture. -: Not applicable

Most students in the survey were enrolled in boarding schools due to the county compulsory education system. The students’ parents worked and lived in rural areas that were usually distantly located from the town or city where the school was located. The students were allowed to go home during school holidays or occasionally on weekends. In Tibetan areas, the government of the Qinghai Province supports all the costs of managing the schools, including student accommodations and living and educational expenses. The poor sanitary conditions and hygiene practices in the school dormitories and the lack of access to clean water are conditions that favor the transmission of parasitic and other infections.

### Ethics statement

Written ethical approval for the study was obtained from the human ethics committee of the Qinghai Province Institute of Endemic Disease Prevention and Control. Formal permission was obtained from the local Education Bureau of Yushu and Guoluo to screen all the school children in the study area. The teachers and parents were informed about the procedures and were assured of free diagnosis and albendazole treatment for any student identified with echinococcosis infection before the commencement of the survey. Consent was obtained from the parents or legal guardians of all the student participants.

### School survey

The survey was conducted in 65 schools between 2011 and 2012. A total of 19 629 students (males: 9954, 50.7%; females: 9675, 49.3%) aged 6–18 years underwent an abdominal examination using a portable ultrasound (Aloka SSD-500, Hitachi Aloka Medical, Ltd., Japan). If suspicious lesion(s) were found in the organs, an additional examination was conducted by another operator using a color ultrasound (Terason 2000, Teratech Corporation, USA) to identify and differentiate between AE and CE patients. Of the surveyed students, 1254 (6.4%) were > 15 years of age, and 18 375 (93.6%) were < 15 years of age. Tibetan students accounted for 94.8% (18 614) of the sample, while the rest were Han Chinese students. All personal details of the students were obtained from the school registration system. After abdominal ultrasound scanning, which involved not only the liver but also the kidneys, spleen, pancreas and other organs in the abdominal cavity, 2–5 ml of venous blood was collected from all the compliant students for serological testing. In total, 16 969 (86.4%) participants were screened by both ultrasound and serology. Serum samples were obtained by standard centrifugation and stored at − 20 °C. The serum samples were used to determine the levels of anti-echinococcosis antibodies.

### Ultrasonography

A detailed description of abnormal lesions or cysts identified in the abdominal cavity during the ultrasound scanning was recorded (including the location, size and any other specific features, such as calcification or liquefaction due to necrosis). All the images were copied and retained on compact disks or universal serial bus storage devices for future reference. Lesions or cysts due to echinococcosis were assessed according to the WHO-Informal Working Group on Echinococcosis (WHO-IWGE) classification for CE or AE [9]. After the ultrasound, the students identified with positive cases of echinococcosis, their parents or guardians and the school principals attended an informative meeting about echinococcosis treatment (surgery where available and/or accessibility to free albendazole) and regular follow-up. Treatment and support were provided by the Chinese central government and delivered by the local Center for Disease Control and Prevention (CDC).

### Serology

The collected human sera were analyzed using a commercial enzyme-linked immunosorbent assay (ELISA) kit (Shenzhen Kangbaide Biotech Co., Ltd., China, lot No: 20120630), provided by the Chinese national control program to qualitatively determine the level of anti-echinococcosis IgG antibodies [10]. Briefly, 100 μl of diluted human serum (1:100) was added in duplicate to ELISA plate microtiter wells precoated with sheep hydatid cyst fluid antigen. The plate was incubated at 37 °C for 30 min. Positive (one well), negative (two wells) and blank (one well) controls were also prepared for each plate. After discarding the contents and washing 3 times with buffer, 50 μl of anti-human antibodies was added to each well, and the plate was incubated at 37 °C for 30 min in the dark. Then, 100 μl of freshly prepared substrate was added to each well, and the plate was incubated at 37 °C until color development. Stop solution was added to each well, and the plate was incubated for 15 min to stop the reaction; the plate absorbance was then read (dual absorption; M: 405 nm, R: 655 nm). The serum samples were considered positive when the absorbance value was higher than or equal to the cut-off value of 3.1 times the average negative control optical density value.

### Statistical analysis

Epi-Info software version 6.0 (Centers for Disease Control and Prevention, Atlanta, GA, USA) was used to record and analyze the survey data. Differences between the groups were compared using the chi-square test.

## Results

### Geographic distribution of childhood echinococcosis cases

The descriptive data of the schools and participants included in the survey and the prevalence rates of AE and CE are summarized in Table 1. A total of 341 students (1.7%, 341/19629) were identified by ultrasound as having either CE (119, 0.6%) or AE (222, 1.1%); only 1 student showed both clinical forms of the infection.

Among the 65 schools surveyed, 9 (3 in Yushu and 6 in Guoluo) did not have any AE or CE cases, 10 (7 in Yushu and 3 in Guoluo) had only CE cases and 11 (2 in Yushu and 9 in Guoluo) had only AE cases. However, the majority of schools (6 in Yushu and 29 in Guoluo) had both AE and CE cases.

In the analysis at the township level, the CE prevalence ranged from 0 to 2.9% (3/105), with the highest rate occurring in Xiahongke Township. The AE prevalence ranged from 0 to 12.1% (25/207), with the highest rate occurring in Tehetu Township. These two townships are located in Dari County, Guoluo Prefecture. Comparing the counties, the highest rates of CE and AE occurred in Gande and Dari counties in Guoluo Prefecture, with prevalence rates of 1.3% (31/2449) and 4.3% (107/2461), respectively. Although there was no significant difference (*P* = 0.08) in CE prevalence across counties, a significant difference (*P* = 0.0003) in AE prevalence was found among six counties in Yushu Prefecture. In Guoluo Prefecture, the differences between the AE prevalence (*P* <  0.001) and CE prevalence (*P* = 0.0012) among the 6 counties were statistically significant. At the prefecture level, the prevalence of AE (1.6%, 191/12175) among Tibetan children in Guoluo was significantly higher than that (0.4%, 31/7454) in Yushu (*P* <  0.001). However, there was no statistically significant difference in CE prevalence between Guoluo (0.8%, 92/12175) and Yushu (0.4%, 27/7454).

### The distribution of echinococcosis child cases by gender and age

A comparison of the total prevalence of AE determined by ultrasound in Yushu and Guoluo revealed a significant difference (*P* <  0.001) between females (1.3%, 130/9675) and males (0.9%, 92/9954) (odds ratio values of 1.1–1.9, mean = 1.4) with a 95% confidence interval (*CI*). Similarly, a significant difference in AE prevalence between males and females was observed only in Guoluo (*P* = 0.013; *OR* = 1.5, 95% *CI*: 1.1–1.9) but not in Yushu (Table 2). In contrast, the analysis results did not show a significant difference in CE prevalence between genders. Although the total AE prevalence did not increase with age (*P* = 0.35), a highly significant difference in the total CE prevalence was evident between younger and older students (*P* <  0.001) (Table 2).

**Table 2:** The distributions of morbidity and seropositive rates in school children by gender/age

Item	Yushu	Guoluo	Yushu & Guoluo^a^			
	Females/Males	Females/Males	≤ 8 years	9–11 years	12–14 years	≥ 15 years
US-scan individuals	3600/3854	6075/6100	3415	8922	6038	1254
AE^a^cases	17/14 (0.5/0.4)	113/78(1.9/1.3)	34(1.0)	94(1.1)	77(1.3)	17(1.4)
CE cases	10/17(0.3/0.4)	48/44(0.8/0.7)	12(0.4)	44(0.5)	50(0.8)	13(1.0)
Echinococcosis -cases	27/31(0.8/0.8)	161/122(2.7/2.0)	46(1.3)	138(1.5)	127(2.1)	30(2.4)
*P*-value	< 0.001		< 0.001			
Sero-test individuals	3422/3659	4984/4909	3087	8225	4772	885
Sero-positive	158/144(4.6/3.9)	942/893(18.9/18.2)	424(13.7)	1121(13.6)	523(11.0)	69(7.8)
*P*-value	< 0.001		< 0.001			

AE: Alveolar echinococcosis; CE: Cystic echinococcosis; US: Ultrasound

^a^AE cases included one case with coexistence of both AE and CE

### Seropositive students for echinococcosis

Serum samples from 16 969 students in 11 counties (no sera from Zaduo County were collected) were tested by ELISA for anti-echinococcosis IgG antibodies (Table 1). The positivity rates ranged from 0.7% (2/276) in Xiewu Township, Chengduo County and Yushu Prefecture to 45.1% (60/133) in Xiadawu Township, Maqin County and Guoluo Prefecture. In general, the mean seroprevalence was 12.6% (2137/16969). At the county level, the highest seropositive rate (23.0%, 429/1864) occurred in Dari County, Guoluo Prefecture, while the lowest seropositive rate (2.2%, 34/1544) was observed in Nangqian County, Yushu Prefecture. Seropositive rates were significantly different between Guoluo and Yushu (*P* <  0.001) and among counties in these two prefectures (*P* <  0.001). Although there were no significant differences in the seropositive rates between males and females, the results revealed that the seropositive rate of echinococcosis increased with age (Table 2).

### Ultrasound classification of AE and CE lesions

In total, 341 cases of echinococcosis were identified by ultrasound in the 2011–2012 surveys (Tables 1 and 2). From these cases, 119 were classified as CE and 222 as AE. A total of 330 cases were assessed by the WHO-IWGE and WHO-IWGE PNM classification systems, and 11 cases were incompletely recorded (3 CE, 8 AE) (Table 3). With respect to CE cases, most lesions were classified as CE1 (45.7%, 53/116), followed by CE3 (37.9%, 44/116), CE2 (10.3%, 12/116) and CE4 (6.0%, 7/116); no CE5 cases were found (Table 3). There were no significant differences between the numbers of early (CE1 and CE2, 65) and mid-stage (CE3, 44) cases, but there was a significant difference between the numbers of early (CE1 and CE2, 65) and later stages (CE4 and CE5, 7) (*P* <  0.001) and between mid-stage and later stages (*P* <  0.001).

**Table 3:** Ultrasound classification of alveolar echinococcosis (AE) and cystic echinococcosis (CE), with lesion anatomic location and lesion numbers amongst school children

Ultrasound cases	Number (%)	Lesion location, *n* (%)			Lesion numbers, *n* (%)			Lesion size, *n* (%)		
		L	R	L + R	1	2	≥ 3	< 5 cm	5–9 cm	≥ 10 cm
CE total	116	25 (21.6)	68 (58.6)	23 (19.8)	86 (74.1)	22 (19.0)	8 (6.9)	39 (33.6)	61 (52.6)	16 (13.8)
CE 1	53 (45.7)	10 (8.6)	32 (27.6)	11 (9.5)	39 (33.6)	11 (9.5)	3 (2.6)	18 (15.5)	27 (23.3)	8 (6.9)
CE 2	12 (10.3)	2 (1.7)	8 (6.9)	2 (1.7)	9 (7.8)	2 (1.7)	1 (0.9)	1 (0.9)	8 (6.9)	3 (2.6)
CE3	44 (37.9)	11 (9.5)	24 (20.7)	9 (7.8)	33 (28.4)	8 (6.9)	3 (2.6)	17 (14.7)	22 (19.0)	5 (4.3)
CE 4	7 (6.0)	2 (1.7)	4 (3.4)	1 (0.9)	5 (4.3)	1 (0.9)	1 (0.9)	3 (2.6)	4 (3.4)	0
CE 5	0	0	0	0	0	0	0	0	0	0
*P*-value	< 0.001	< 0.001			< 0.001			< 0.001		
AE total	214	45 (21.0)	133 (62.1)	36 (16.8)	153 (71.5)	38 (17.8)	23 (10.7)	187 (87.4)	21 (9.8)	6 (2.8)
P 1 a^+^	145 (67.8)	34 (15.9)	111 (51.9)	0	122 (57.0)	19 (8.9)	4 (1.9)	145 (67.8)	0	0
P1 b^++^	23 (10.7)	0	0	23 (10.7)	0	12 (5.6)	11 (5.1)	23 (10.7)	0	0
P2	35 (16.4)	11 (5.1)	22 (10.3)	2 (0.9)	27 (12.6)	5 (2.3)	3 (1.4)	18 (8.4)	15 (7.0)	2 (0.9)
P3	10 (4.7)	0	0	10 (4.7)	3 (1.4)	2 (0.9)	5 (2.3)	1 (0.5)	6 (2.8)	3 (1.4)
P4	1 (0.5)	0	0	1 (0.5)	1 (0.5)	0	0	0	0	1 (0.5)
*P*-value	< 0.001	< 0.001			< 0.001			< 0.001		

L: Left liver lobe; R: Right liver lobe; L + R: Both liver lobes including left and right. P1 a^+^: P1 type lesion located in one liver lobe; P1 b^++^: Several P1 type lesions located in both lobes

Most of the lesions were located in the right liver (58.6%, 68/116); lesions were also found in the left liver (21.6%, 21.6/116) and in both liver lobes (19.8%, 23/116) (*P* <  0.001). Most of the CE cases (74.1%, 86/116) had a single lesion, but there were also cases with 2 (19.0%, 22/116) or > 2 lesions (6.9%, 8/116) (*P* <  0.001). In general, the most common lesions were medium-sized (52.6%, 61/116), followed by the small-sized (33.6%, 39/116) and large-sized (13.8%, 16/116).There were significant differences between the numbers of small- and large-sized lesions (*P* <  0.001) and between the numbers of medium- and large-sized lesions (*P* <  0.001).

Regarding the AE cases, the very early P1 stage accounted for 78.5% (168/214) of the cases, followed by the P2 (16.4%, 35/214) and P3 stages (4.7%, 10/214); only 1 case was recorded as the P4 stage. There were significant differences in the frequency of cases with very early P1 compared with early P2 stage lesions (*P* <  0.001) and between early P1/P2 and advanced P3/P4 stage lesions (*P* <  0.001). Most of the AE cases involved lesions in the right liver lobe (62.1%, 133/214). The prevalence of the location of right liver lobe lesions was significantly different from those of the other 2 locations (*P* <  0.001). Single lesions accounted for 71.5% (153/214) of the AE cases, and 2 lesions for 17.8% (38/214) and ≥ 3 lesions accounted for 10.7% (23/214) (Table 3). The lesions were predominantly small-sized, accounting for 87% (187/214) of the cases, followed by medium-sized lesions (9.8%, 21/214) and large-sized (2.8%, 6/214). The differences between the number and the size of the lesions were significant (*P* <  0.001).

## Discussion

The major ethnic group in Yushu and Guoluo is Chinese-Tibetan. Families in this group have a nomadic lifestyle and undertake traditional agricultural work and animal husbandry as their major source of income. Typically, Tibetans are on the move from spring to autumn to search for better pasture for their livestock, which includes mainly yaks and sheep. Tibetans also keep 2 or more dogs for herding and guarding purposes. These dogs spend most of their time unrestrained and roaming, especially at night. The Tibetan families live in tents and move according the grass and river (as main water supply). So they cannot keep the living conditions as good as peoples living in the city. Tibetan groups commonly perform home slaughter of sheep and other livestock for family meat consumption. Overgrazing of grassland pastures by their livestock has resulted in highly favorable new environments for increased numbers of small mammals, including many susceptible species that can act as intermediate hosts for *E. multilocularis* [8, 11]*.* Roaming Tibetan dogs can scavenge for these small mammals [8, 12], and readily access *E. granulosus*-infected offal or viscera that may be discarded as a result of the practice of home slaughter [13]. Traditionally, based on Buddhist beliefs, Tibetans are encouraged to feed stray dogs, which results in a considerable increase in the number of stray dogs not only in and around the temples but also in heavily populated locations such as schools, hospitals, entertainment parks and open markets. This large concentration of roaming stray dogs, coupled with poor sanitation and hygiene practices, provides an ideal environment for *Echinococcosis* transmission. Although hyper-endemicity of CE and AE on the Tibetan Plateau has been well documented for the past decade [13, 14], most reports only considered adult cases of echinococcosis, with limited discussion about child morbidity due to echinococcosis. The lack of baseline data on echinococcosis morbidity and *Echinococcus* exposure rates in children has hampered an accurate epidemiological evaluation of the prevalence of the disease in this group, which is an important consideration for the implementation of control strategies and policy development.

The active search for asymptomatic human cases of AE and CE is widely recognized as an effective method for improving patient prognosis and defining the epidemiological profile of echinococcosis in any endemic area. This type of search is also a necessary step for accurately assessing the true extent of the disease in a region. Through active surveys, this study, which was undertaken in the previously recognized hyper-endemic area of the Tibetan Plateau [8, 11, 13, 14], revealed a high prevalence of CE and AE among Tibetan children. This outcome provided the opportunity for early treatment of infected young participants and offered strong supporting evidence concerning the occurrence and transmission of both types of echinococcosis during childhood, for which only scarce data are currently available [15, 16]. The lack of morbidity data for AE and CE in children has made the introduction of preventive measures difficult worldwide, including in China. Echinococcosis is mainly diagnosed in adults and rarely in children because it tends to grow slowly, with a long-term asymptomatic period (generally up to 15 years) followed by chronic and then advanced stages that become evident when symptoms commence. Even in patients in the asymptomatic period, the disease course is progressive but slow, and there is a typical development of liver pathology. In many cases after the initial diagnosis, the disease may progress to a critical stage if left untreated [1, 17].

Serological testing is useful for not only diagnosis but also detecting exposure to the parasite before the clinical manifestations of the disease develop [10]. Therefore, the failure to identify seropositive individuals by ultrasound suggested either an early stage of echinococcosis that lacks evident hepatic changes or individual resistance to echinococcosis infection. In this study, there were no gender-related differences in the seropositive rates between children, suggesting equal exposure to echinococcosis egg-contaminated environments [18]. In general, the cases of echinococcosis identified by serology and ultrasound seemed to increase with age. The seropositive rate increased from the age of 9 years, and the cases identified by ultrasound increased from the age of 12 years. These findings reflect the incubation time after the ingestion of eggs (i.e., exposure). The results also showed that the prevalence of echinococcosis varied across counties and schools in this region, which may suggest that there are specific local factors that determine the different levels of contamination with *Echinococcus* eggs. Based on the ultrasound reports, the prevalence rates of CE and AE ranged from 0 to 12%. These findings indicate that there is no transmission to humans in some areas and suggest that some townships lack efficient echinococcosis control programs, such as the dog-deworming campaign launched by the Chinese Central Government several years ago [19].

Although the findings of this study indicate that the transmission of both *E. granulosus* and *E. multilocularis* is very active in Yushu and Guoluo, there were some clear limitations regarding the accurate estimation of echinococcosis prevalence in both prefectures. First, the results likely underreport the true prevalence of echinococcosis in these regions. This study incorporated only abdominal ultrasound scanning and may have missed children with lesions in other organs, particularly children with CE, which frequently affects multiple organs in younger populations [20–22]. Additionally, some reports have suggested that the cysts from CE in children are more frequently located in the lungs than in the liver [20]. Second, the ELISA-based serological test kit used in this study does not differentiate AE from CE cases. Although this test has high sensitivity for detecting anti-*E. granulosus* antibodies, there may be high cross-reactivity with *E. multilocularis* antigens and other antigens from the cestode species family [23]. As a result, the exact determination of the transmission of echinococcosis in children becomes challenging, despite indications for the potential co-existence of both types of echinococcosis [10, 23]. Additionally, this survey was inadequate because the sample of children may not have been representative of the general population; therefore, to understand the prevalence of echinococcosis, further investigations should be performed in different groups on the Qinghai-Tibetan Plateau.

## Conclusions

The findings of the present study suggest that the Qinghai Province is a highly endemic area for echinococcosis, where children are commonly at risk. Echinococcosis prevention and control measures on the Tibetan Plateau have potentially failed due to a poor understanding of the epidemiology of this infection in the region. These results provide some indication that the local harsh natural environment, the lower level of economic development in the plateau area, the poor hygienic practices and the lack of knowledge about echinococcosis transmission are important determinants in infection transmission. Therefore, a comprehensive program targeting echinococcosis should focus on not only strategies to improve early diagnosis and treatment but also educational campaigns to improve awareness about the disease and the sanitation and hygienic practices among traditional Tibetan families.

## Additional file

Additional file 1: Multilingual abstracts in the six official working languages of the United Nations. (PDF 625 kb)

## Abbreviations

AE: Alveolar echinococcosis
CDC: Center for Disease Control and Prevention
CE: Cystic echinococcosis
DALYs: Disability-adjusted life years
TAPs: Tibetan Autonomous Prefectures
US: Ultrasound
WHO-IWGE: WHO-Informal Working Group on Echinococcosis
