# Supplementary material for: Childhood suffering: hyper endemic echinococcosis in Qinghai-Tibetan primary school students, China
Source: Infect Dis Poverty. 2018 Jul 12;7:71. doi: 10.1186/s40249-018-0455-y (PMC6042437; doi:10.1186/s40249-018-0455-y)
Supplement: Supplementary file 1 — Multilingual abstracts in the six official working languages of the United Nations. (PDF 625 kb) [file 40249_2018_455_MOESM1_ESM.pdf]

Translation of the abstract into the six official working languages of the United Nations

معاناة الطفولة: داء المشوكات شديد التوطن في تشينغهاي - بين تلاميذ المدارس الابتدائية التبتيين، بالصين .

شيو مِن هَن، تشي جانج تساي، وي وُنج، هو وُنج، كيانج تشانج , يونج شون وانج

الملخص

الخلفية: كجزء لا يتجزأ من برنامج مستمر يهدف إلى استخدام الاكتشاف المبكر والعلاج الفوري، لتحسين مكافحة داء المشوكات خاصة في الفئات العمرية الأصغر سناً، قمنا بعمل سلسلة من الدراسات النشطة بين التلاميذ التبتيين في مقاطعة تشينغهاي شمال غرب الصين في عامي 2011 و 2012. وتؤكد النتائج المهمة التي نشأت عن هذه الدراسة على ضرورة لفت الانتباه إلى داء المشوكات، لكل من داء المشوكات السنخي (AE)، وداء المشوكات الكيسي (CE) على حد سواء، حتى تُعَزَز خطة التطوير وتُحدّد الوسائل المناسبة للمكافحة في المناطق الموبوءة للغاية على هضبة التبت.

الطرق: إجمالي 19629 تلميذ إبتدائي، أعمارهم من 6- 18 عام، لديهم ثقافة تبتية سائدة، خضعوا لفحص موجات فوق صوتية للبطن. وقد تبرع 86.4% من التلاميذ الخاضعين للفحص ب 2-5 مل من الدم الوريدي لإجراء الاختبارات المصلية. وسُجلت كل النتائج غير الطبيعية لفحص الموجات فوق الصوتية. في حالة تم التشخيص بأنه داء المشوكات، فإن ضرر المرض يقّيَم وفقاً لفريق العمل غيرالرسمي التابع لمنظمة الصحة العالمية (IWGE-WHO) على تصنيف داء المشوكات السنخي AE و داء المشوكات الكيسي CE. تم عقد مقارنة بين معدل انتشار المرض في المدرسة، وبين المواقع الجغرافية والجنس والفئات العمرية للتلاميذ الذين خضعوا للدراسة. حُلِلت عروض الصور السريرية وفقا لكمّ الآفة وحجمها وموقعها في الكبد ومرحلة التصنيف. حُدِّدَت الأهمية الإحصائية عند القيمة الاحتمالية < 0.05 للمقارنة بين المجموعات.

النتائج: إجمالي 341 تلميذا (1.7%) تم التعرف على إصابتهم إما بداء المشوكات الكيسي (119, 0.6%) أو داء المشوكات السنخي (222, 1.1%) عن طريق فحص الموجات فوق الصوتية. وقد ظهرت أعلى معدلات انتشار لداء المشوكات السنخي في الطفولة، في بلدتي تيهيتو (12.1%) وموبا (11.8%) بإقليم داري. وكانت هناك معدلات مرتفعة للحالات الإيجابية مصلياً (37.0%)، وتوزيع غير متجانس للحالات، مع معدلات انتشار تتراوح بين 0-12.1% لداء المشوكات السنخي، 0-2.9% لداء المشوكات الكيسي. فضلاً عن ذلك ، تراوحت معدلات الحالات الإيجابية مصلياً من 0.7-45.1% عبر مختلف المدارس.

الاستنتاجات: التفشّ الواسع لداء المشوكات في تشينغهاي - بين تلاميذ المدارس الابتدائية التبتيين- يعكس نقص المعرفة بشأن طرق انتقال أنواع المشوكات. ويمكن للمزج بين التعليم النظامي للأطفال، والقيام بإجراء علاج الكلاب من الديدان بانتظام، تحقيق هدف المكافحة المستدامة للعداري.

Translated from English version into Arabic bySusan Fegy, proofread by Free bird, through

TRANSLATORS WITHOUT BORDERS

童年苦痛：包虫病在中国青藏高原小学生中呈高度地方流行性

韩秀敏，蔡其刚，王威，王虎，张强，王永顺

摘要

引言：一项正在进行的项目是通过早期检测和及时治疗（尤其是对幼龄儿童）以提高对包虫病的控制。作为该项目的一部分，我们于2011–2012年对中国青海省的藏族儿童进行了一系列主动病例侦查。该研究主要结果强调泡型包虫病（AE）和囊性包虫病（CE）应引起人们的足够重

视，以促进包虫病在呈高度地方流行的青藏高原地区的政策制定及其确定最适控制途径。
方法：对19 269名年龄在6–18岁的藏族小学生进行腹部超声检查，收集其中86.4％小学生的静脉血(2–5 ml)作血清学检测。记录所有异常超声检测结果。如鉴定为包虫病，根据世界卫生组织棘球蚴病非正式工作组（WHO-IWGE）对AE和CE的分类标准评估疾病的损害程度。在被调查的学生中，按地理位置、性别和年龄分组比较学校间的流行率。根据病灶数量、大小、在肝脏中的位置和分类阶段分析临床影像特征。设置P值<0.05时，组间比较具有统计学意义。
结果：超声检测共确定了341名小学生（1.7％）患包虫病，其中囊型包虫病119例（0.6％），泡型包虫病222例（1.1％）。儿童泡型包虫病患病率最高的地区是达日县的特合土乡（12.1％）和莫坝（11.8％）乡。血清阳性率高（37.0％）且病例分布不均匀，其中泡型包虫病血清阳性率为0–12.1％，囊型包虫病血清阳性率为0–2.9％。此外，不同学校的血清阳性率为0.7–45.1％。
结论：青海藏族小学生包虫病的高发病率反映出对棘球绦虫传播的认识不足。对儿童进行系统教育、定期对狗进行驱虫，两者相结合可达到持续控制包虫病的目的。

Translated from English version into Chinese by Qi-Gang Cai

Souffrances enfantines : l'échinococcose hyperendémique parmi les écoliers tibétains des écoles primaires du Qinghai, en Chine

Xiu-Min Han, Qi-Gang Cai, Wei Wang, Hu Wang, Qiang Zhang, Yong-Shun Wang

Résumé
Contexte: Dans le cadre d'un programme permanent de dépistage et de traitement précoce de l'échinococcose visant à améliorer la lutte contre cette parasitose, en particulier parmi les classes d'âge les plus jeunes, nous avons entrepris en 2011 et 2012 une série d'enquêtes de terrain parmi les enfants tibétains de la province du Qinghai, dans le nord-ouest de la Chine. Les résultats significatifs de cette étude soulignent la nécessité d'attirer l'attention sur l'échinococcose, aussi bien alvéolaire que kystique, afin de promouvoir l'élaboration de politiques et d'identifier les méthodes de lutte qui conviendraient pour les régions de forte endémie du plateau tibétain.
Méthodes: Des échographies abdominales ont été pratiquées sur 19 629 écoliers âgés de 6 à 18 ans, d'ascendance principalement tibétaine. Un échantillon de 2 à 5 ml de sang veineux a également été prélevé chez 86,4 % des enfants retenus en vue de tests sérologiques. Tous les résultats d'échographie anormaux sont consignés dans le tableau 4. Les anomalies identifiées comme des lésions d'échinococcose ont été évaluées selon la classification du groupe de travail informel de l'OMS sur l'échinococcose (WHO-IWGE), applicable aux formes alvéolaire aussi bien que kystique. Parmi les écoliers examinés, la prévalence par école a été comparée en fonction de la localisation géographique, du sexe et de la tranche d'âge. Les images cliniques ont été analysées selon le nombre de lésions, leur taille, leur emplacement dans le foie et leur stade. Pour les comparaisons entre groupes, les résultats ont été jugés significatifs si la valeur de P était < 0,05.
Résultats: L'échographie a révélé chez un total de 341 écoliers (1,7 %) une échinococcose kystique (119, 0,6 %) ou alvéolaire (222, 1,1 %). Les taux de prévalence les plus élevés d'échinococcose alvéolaire infantile ont été relevés dans les localités de Tehetu (12,1 %) et Moba (11,8 %), dans la circonscription de Dari. Nous avons constaté un taux élevé de séropositivité (37 %) et une distribution

hétérogène des cas, avec une prévalence comprise entre 0 et 12,1 % pour la forme alvéolaire et entre 0 et 2,9 % pour la forme kystique. En outre, le taux de séropositivité mesuré dans différentes écoles était compris entre 0,7 % et 45,1 %.

Conclusions: La forte prévalence de l'échinococcose parmi les élèves tibétains des écoles primaires du Qinghai reflète le manque de connaissance relatives à la transmission d'*Echinococcus* spp. La combinaison d'une éducation systématique des enfants et de traitements anthelminthiques réguliers des chiens permettrait d'atteindre l'objectif d'un contrôle durable de l'hydatidose.

Translated from English version into French by Gram Matike, proofread by Suzanne Assenat, through

Детские страдания: гиперэндемичный эхинококкоз среди школьников Цинхай-Тибетского нагорья, Китай

Сю-Минь Хань, Ци-Ган Цай, Вэй Ван, Ху Ван, Цян Чжан, Юн-Шунь Ван

Аннотация

Справочная информация: В рамках текущей программы, нацеленной на использование ранней диагностики и своевременного лечения для улучшения контроля за эхиноккокозом, в особенности среди молодых групп населения, в период с 2011 по 2012 гг. нами был проведён ряд активных обследований детей Цинхай-Тибетского нагорья провинции Цинхай на северо-западе Китая. Значимость результатов данного исследования подчеркивает необходимость привлечения внимания как к альвеолярному эхинококкозу, так и к кистозному эхинококкозу, так, чтобы в высокоэндемичных районах Тибетского нагорья продвигалось формирование политики и надлежащих путей контроля за указанными заболеваниями.

Методы: В общей сложности 19 629 школьников в возрасте от 6 до 18 лет с доминантным тибетским происхождением подверглись ультразвуковому обследованию брюшной полости; и 86,4% соответствующих учеников сдали 2–5 мл венозной крови для серологического анализа. Были зарегистрированы все аномальные результаты ультразвукового обследования. В случае выявления эхинококкоза обследование очага поражения производилось в соответствии с классификацией Неофициальной рабочей группы ВОЗ по эхинококкозу (ВОЗ-НРГЭ) по альвеолярному эхинококкозу и кистозному эхинококкозу. Среди учеников, прошедших обследование, сравнение школьной заболеваемости среди групп производилось по географическим координатам, полу и возрасту. Анализ представленной клинической картины был выполнен в соответствии с количеством, размером и расположением очага в печени, а также в зависимости от стадии заболевания. Уровень статистической значимости был установлен на отметке P-значения $< 0,05$ для сравнений по группам.

Результаты: На основании ультразвукового обследования у общего числа 341 школьников (1,7%) был выявлен либо кистозный (119; 0,6%), либо альвеолярный эхинококкоз (222; 1,1%).

Наиболее высокий уровень детской заболеваемости альвеолярным эхинококкозом наблюдался в посёлках Техету (12,1%) и Моба (11,8%) района Дари. Наблюдались высокий серопозитивный уровень (37,0%) и неоднородное распределение случаев заболевания, сопровождающиеся колебанием уровня заболеваемости от 0 до 12,1% по альвеолярному эхинококкозу и от 0 до 2,9% по кистозному эхинококкозу. Более того, уровень серопозитивных результатов анализов колебался от 0,7 до 45,1% по разным школам.

Выводы: Высокая заболеваемость эхинококкозом среди школьников Цинхай-Тибетского нагорья отражает недостаток знаний относительно передачи *особей* рода эхинококка. Сочетание систематического образования детей с регулярным лечением собак противоглистовыми препаратами может способствовать достижению цели устойчивого контроля за однокамерным эхинококкозом.

Translated from English version into Russian by Liudmila Tomanek, proofread by Datran, through

TRANSLATORS
WITHOUT BORDERS

Infancia que sufre: equinococosis hiperendémica en estudiantes de escuelas primarias de Qinghai-Tíbet, China

Xiu-Min Han, Qi-Gang Cai, Wei Wang, Hu Wang, Qiang Zhang, Yong-Shun Wang

Resumen

Contexto: Como parte de un programa continuo que tiene como objetivo utilizar la detección temprana y el tratamiento oportuno para mejorar el control de la equinococosis, en particular en grupos de edades más jóvenes, comenzamos una serie de estudios activos entre niños de Qinghai-Tíbet en la provincia de Qinghai al noroeste de China en 2011 y 2012. Los resultados más significativos de este estudio se centran en la necesidad de poner el foco sobre la equinococosis, tanto la equinococosis alveolar (EA) como la equinococosis quística (EQ), para que se promueva el desarrollo de políticas y se identifiquen los medios adecuados para el control en las áreas endémicas en la meseta tibetana.

Métodos: Un total de 19 629 estudiantes de escuelas primarias, de entre 6 y 18 años de edad, con un origen predominantemente tibetano fue sometido a un examen de ultrasonido abdominal. Además, el 86,4 % de los estudiantes donó, en conformidad, 2-5 ml de sangre venosa para pruebas de serología. Se registraron todos los resultados anormales del ultrasonido abdominal. Si la lesión de la enfermedad se determinaba como equinococosis, se evaluaba de acuerdo con la clasificación para la EA y la EQ del Grupo de Trabajo Informal de la OMS para la Equinococosis (WHO-IWGE, por sus siglas en inglés). Entre los estudiantes que participaron del estudio, se comparó la preponderancia por escuela entre las geolocalizaciones y los grupos según sexo y edad. Se analizaron las imágenes clínicas de acuerdo con el número de lesiones, el tamaño, la ubicación en el hígado y el proceso de clasificación. El significado estadístico se estableció en *valor de p* inferior a 0,05 para las comparaciones entre los grupos.

Resultados: Se identificó por ultrasonido que un total de 341 estudiantes (1,7 %) tenía o EQ (119;

0,6 %) o EA (222; 1,1 %). Los índices más altos de prevalencia de casos infantiles de EAocurrieron en los municipios de Tehetu (12,1%) y Moba (11,8 %) en el condado de Dari. Hubo un alto índice de seropositividad (37,0 %) y una distribución heterogénea de casos, con una preponderancia que varió desde 0 a 12,1 % para la EA y de 0 a 2,9 % para la EQ. Además, el índice de seropositividad varió desde 0,7 a 45,1 % en las diferentes escuelas.

Conclusiones: La alta preponderancia de la equinococosis en los estudiantes de escuelas primarias de Qinghai-Tíbet refleja una falta de información sobre la transmisión de *Echinococcus* spp. La combinación de una educación sistemática para los niños y un tratamiento antihelmíntico que se realice de manera regular en los perros podría ayudar a alcanzar el objetivo de lograr un control sostenible de la hidatidosis.

Translated from English version into Spanish by Maximiliano Juncos, proofread by Maria Paula Gorgone, through

TRANSLATORS
WITHOUT BORDERS
